# Supplementary material for: Xbra modulates the activity of linker region phosphorylated Smad1 during Xenopus development
Source: Sci Rep. 2024 Apr 18;14:8922. doi: 10.1038/s41598-024-59299-7 (PMC11026473; doi:10.1038/s41598-024-59299-7)
Supplement: Supplementary file 1 — Supplementary Figures. [file 41598_2024_59299_MOESM1_ESM.pdf]

Figure: 1.

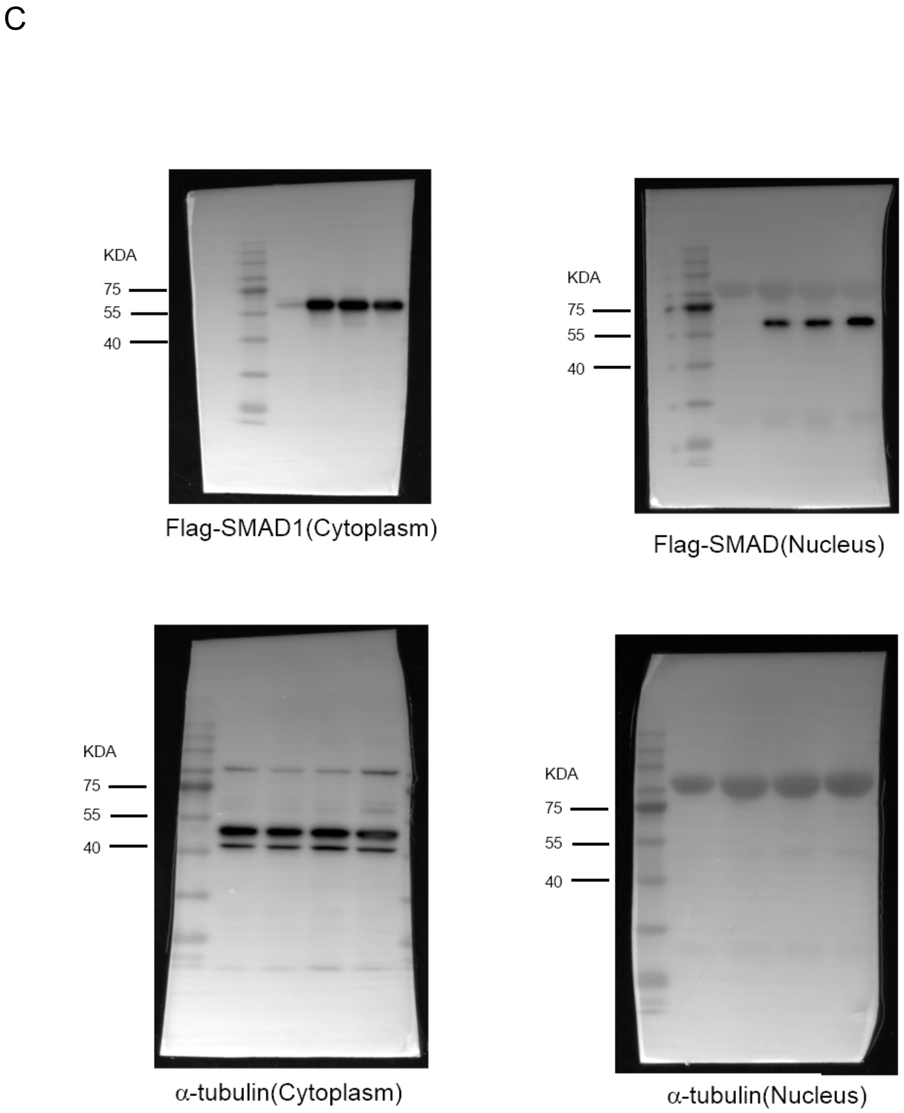
C
KDA
75
55
40
Flag-SMAD1(Cytoplasm)
KDA
75
55
40
Flag-SMAD(Nucleus)
KDA
75
55
40
α-tubulin(Cytoplasm)
KDA
75
55
40
α-tubulin(Nucleus)

Figure: 2

A

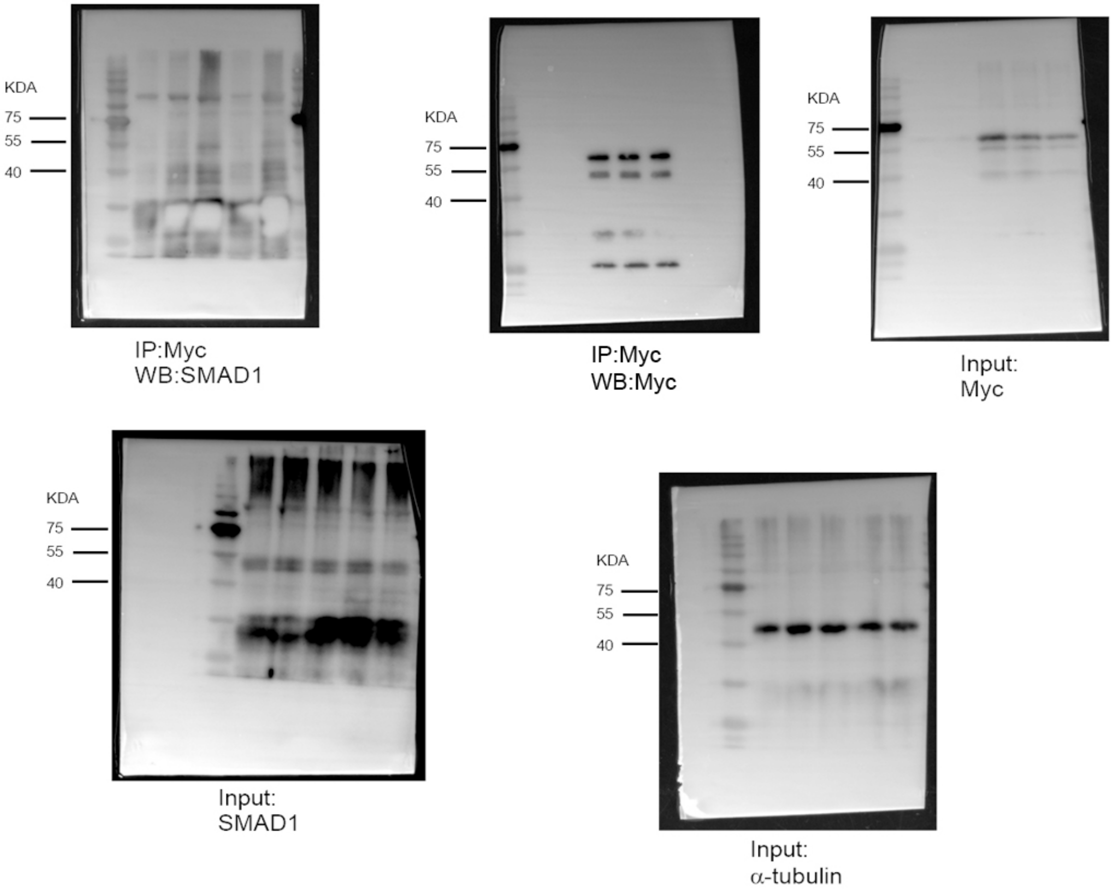
KDA
75
55
40
IP:Myc
WB:SMAD1
KDA
75
55
40
IP:Myc
WB:Myc
KDA
75
55
40
Input:
Myc
KDA
75
55
40
Input:
SMAD1
KDA
75
55
40
Input:
α-tubulin

B

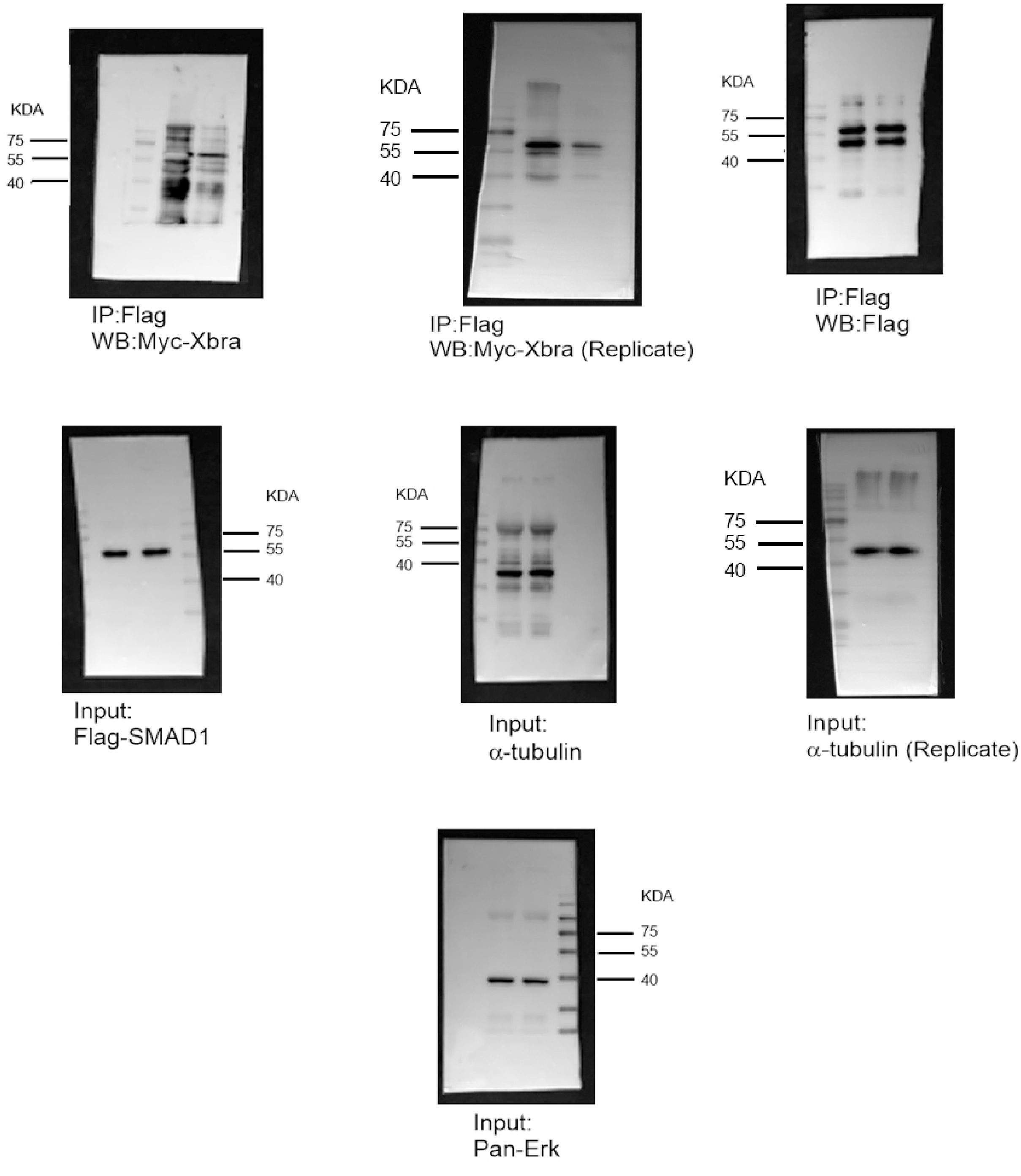
KDA
75
55
40
IP:Flag
WB:Myc-Xbra
KDA
75
55
40
IP:Flag
WB:Myc-Xbra (Replicate)
KDA
75
55
40
IP:Flag
WB:Flag
KDA
75
55
40
Input:
Flag-SMAD1
KDA
75
55
40
Input:
α-tubulin
KDA
75
55
40
Input:
α-tubulin (Replicate)
KDA
75
55
40
Input:
Pan-Erk

C

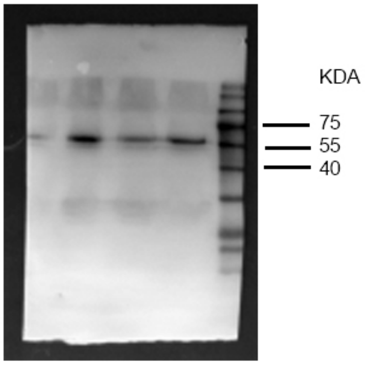

Flag-SMAD1s206

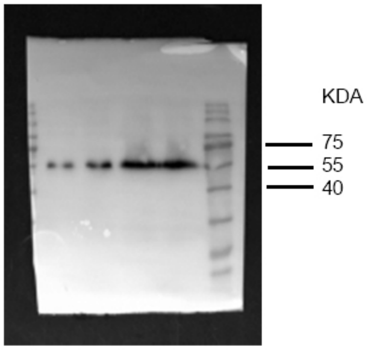

Flag-SMAD1/5/9c-ter.

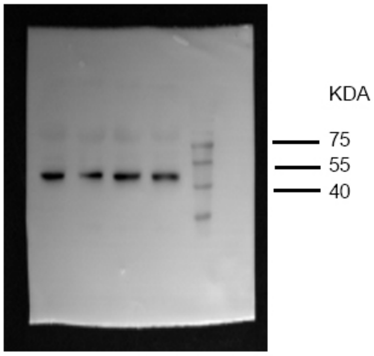

α-tubulin

E

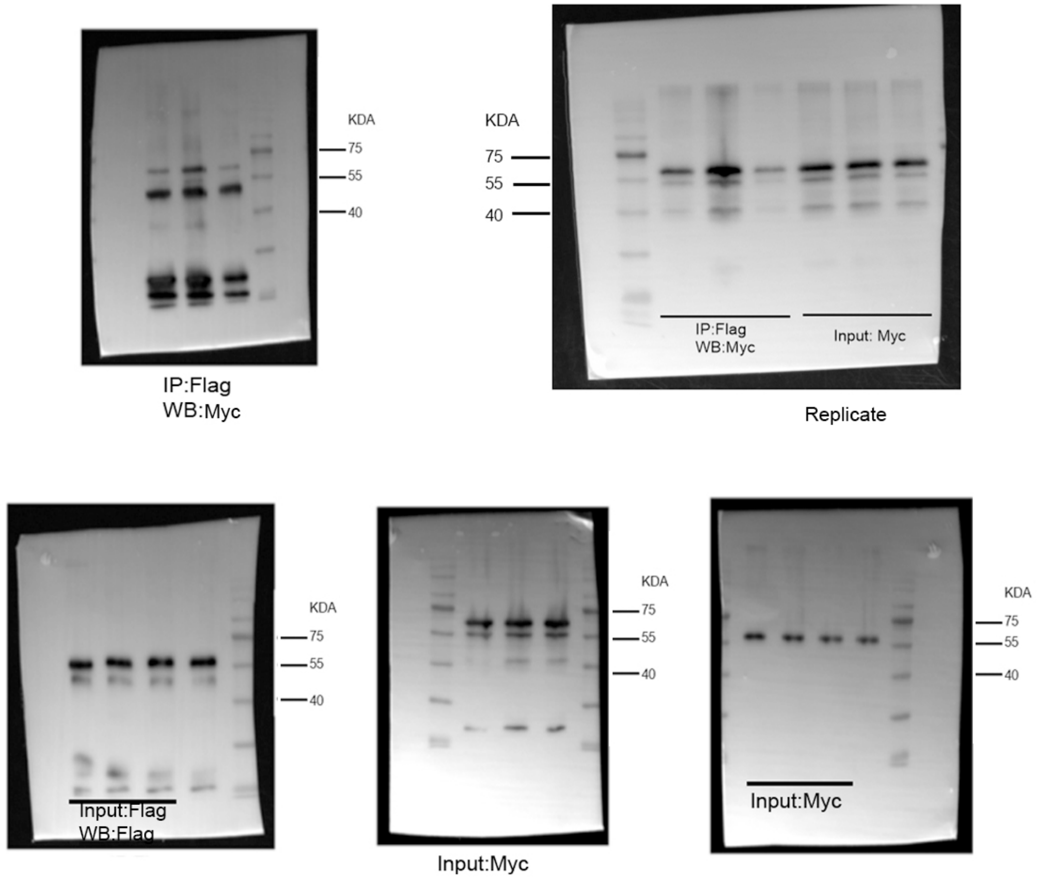
KDA
75
55
40
IP:Flag
WB:Myc
KDA
75
55
40
IP:Flag
WB:Myc
Input: Myc
Replicate
KDA
75
55
40
Input:Flag
WB:Flag
KDA
75
55
40
Input:Myc
KDA
75
55
40
Input:Myc

Figure: 4

A

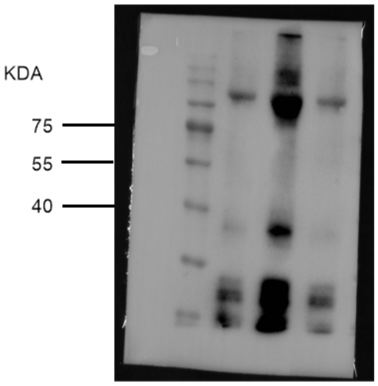

IP:SMAD1
WB:Flag

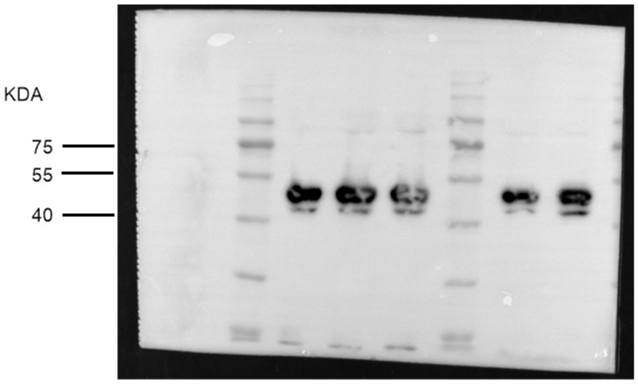

Input:
α-tubulin

B

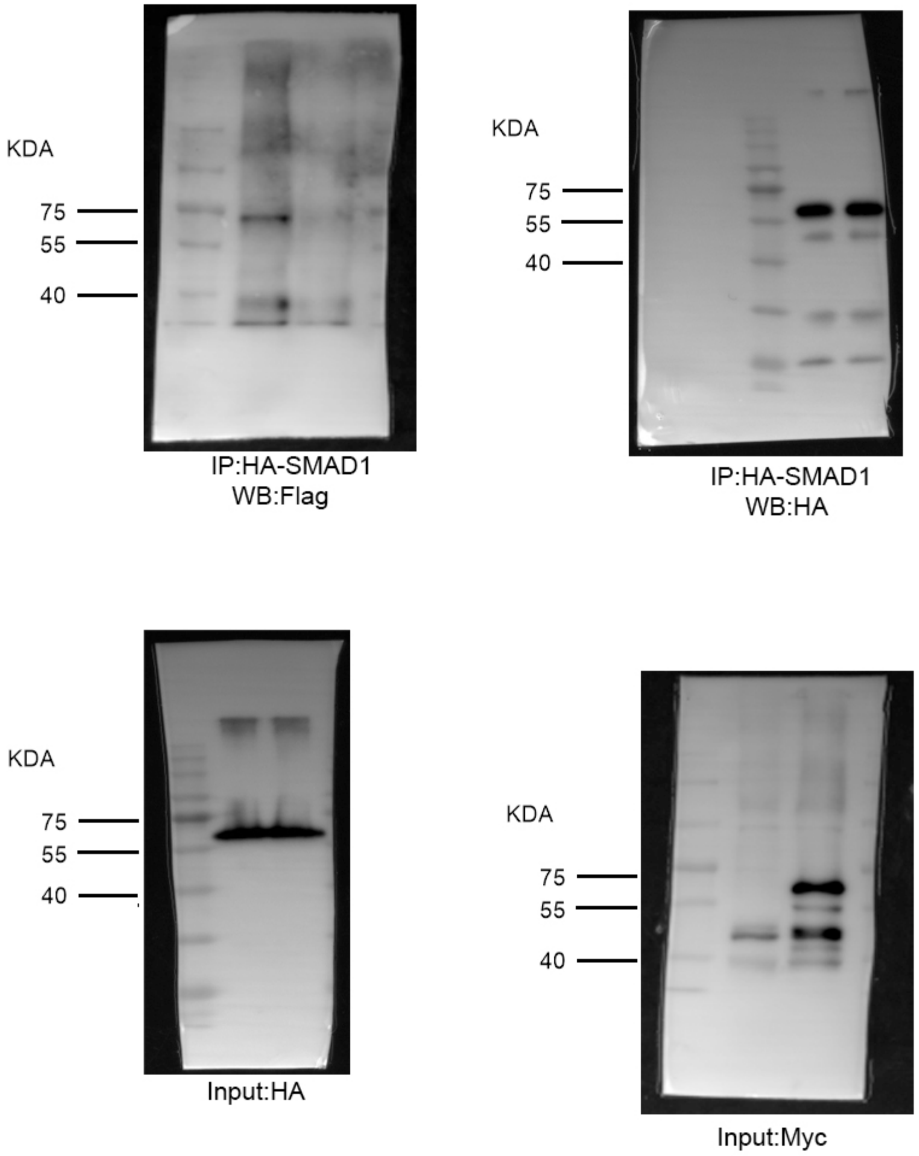
KDA
75
55
40
IP:HA-SMAD1
WB:Flag
KDA
75
55
40
IP:HA-SMAD1
WB:HA
KDA
75
55
40
Input:HA
KDA
75
55
40
Input:Myc

C

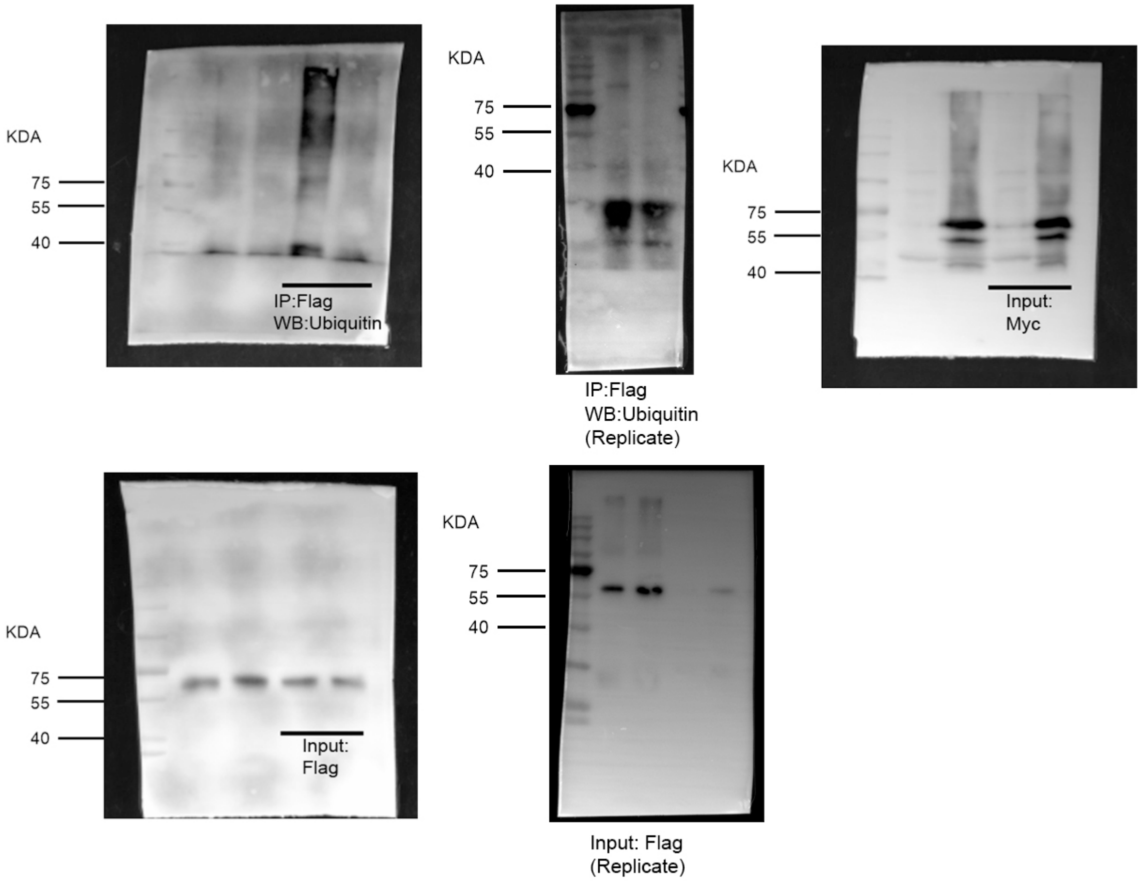
KDA
75
55
40
IP:Flag
WB:Ubiquitin
KDA
75
55
40
IP:Flag
WB:Ubiquitin
(Replicate)
KDA
75
55
40
Input:
Myc
KDA
75
55
40
Input:
Flag
KDA
75
55
40
Input: Flag
(Replicate)

D

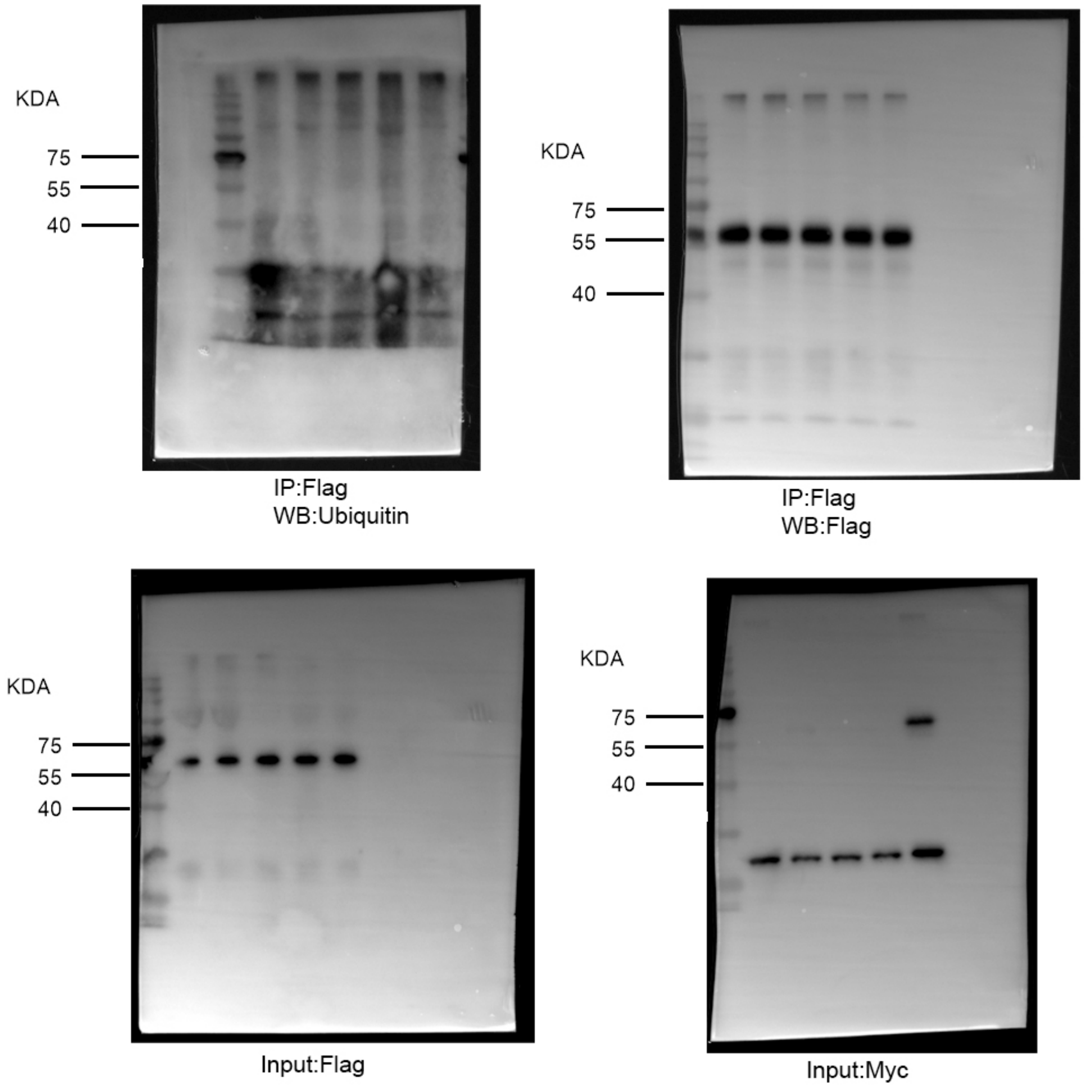
KDA
75
55
40
IP:Flag
WB:Ubiquitin
KDA
75
55
40
IP:Flag
WB:Flag
KDA
75
55
40
Input:Flag
KDA
75
55
40
Input:Myc

E

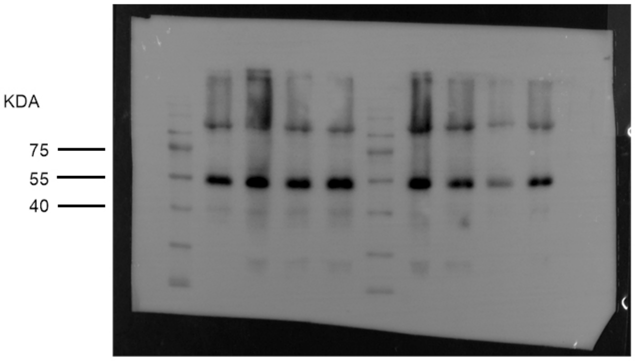

SMAD1
0 Hrs.

SMAD1
3 Hrs.

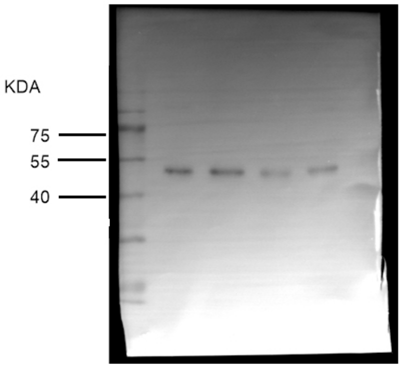

SMAD1
6 Hrs.

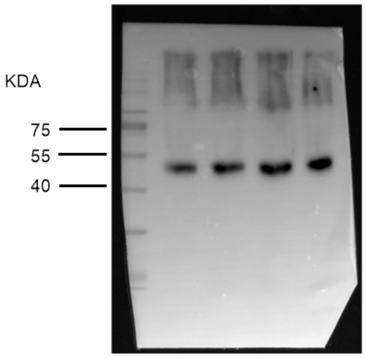

Input:
α-tubulin

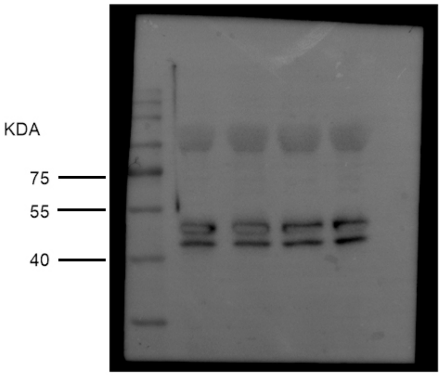

Input:
α-tubulin

F

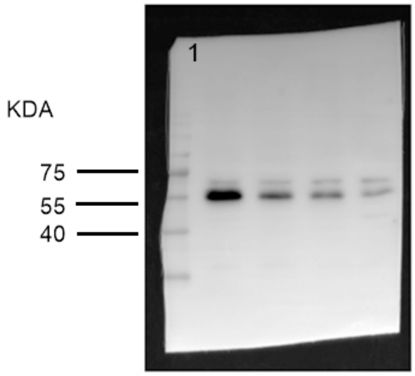

Flag-SMAD1(4SD3SD)

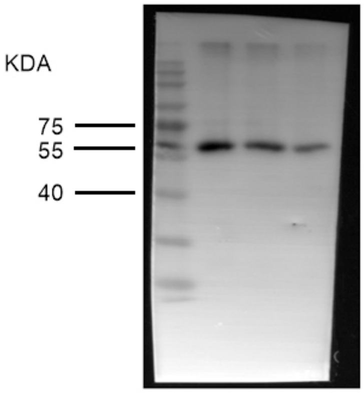

Flag-SMAD1(4SD3SD)
(Replicate)

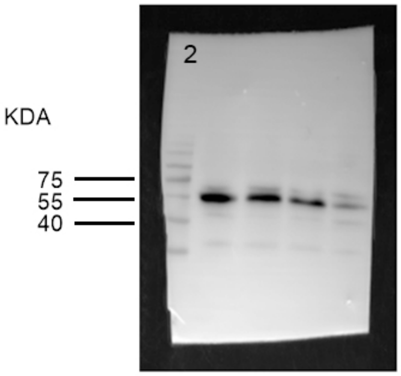

Flag-SMAD1(4SD3SD)
+Xbra

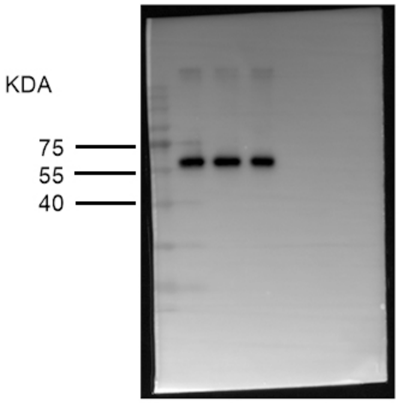

Flag-SMAD1(4SD3SD)
+Xbra (Replicate)

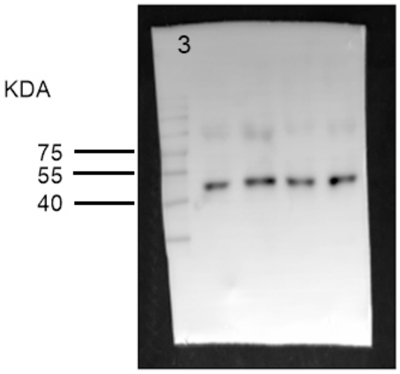

Input:
α-tubulin

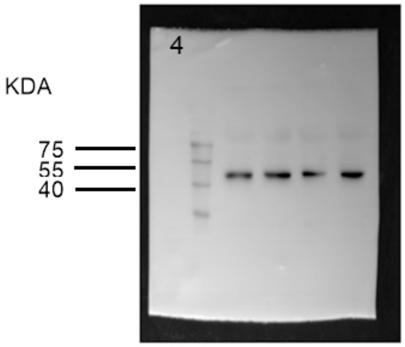

Input:
α-tubulin

In case of 1,2,3 & 4 experiment was done
until 8 Hrs.until 6 Hrs results were used

G

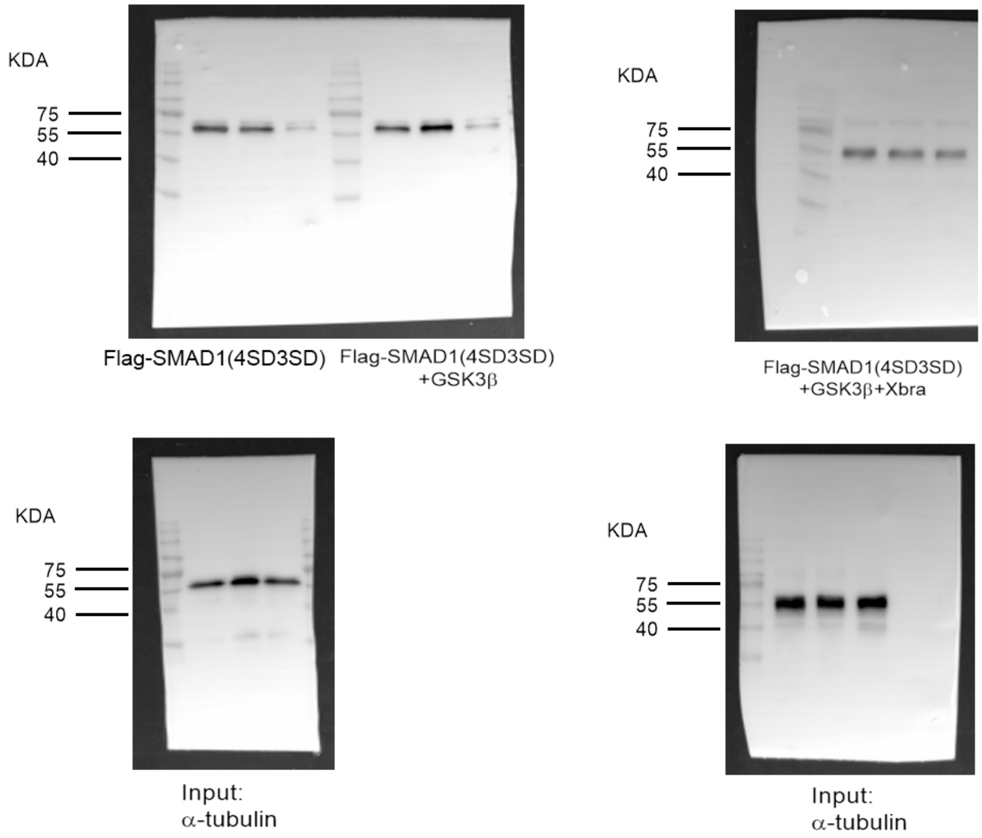
KDA
75
55
40
Flag-SMAD1(4SD3SD)
Flag-SMAD1(4SD3SD)
+GSK3β
KDA
75
55
40
Flag-SMAD1(4SD3SD)
+GSK3β+Xbra
KDA
75
55
40
Input:
α-tubulin
KDA
75
55
40
Input:
α-tubulin

H

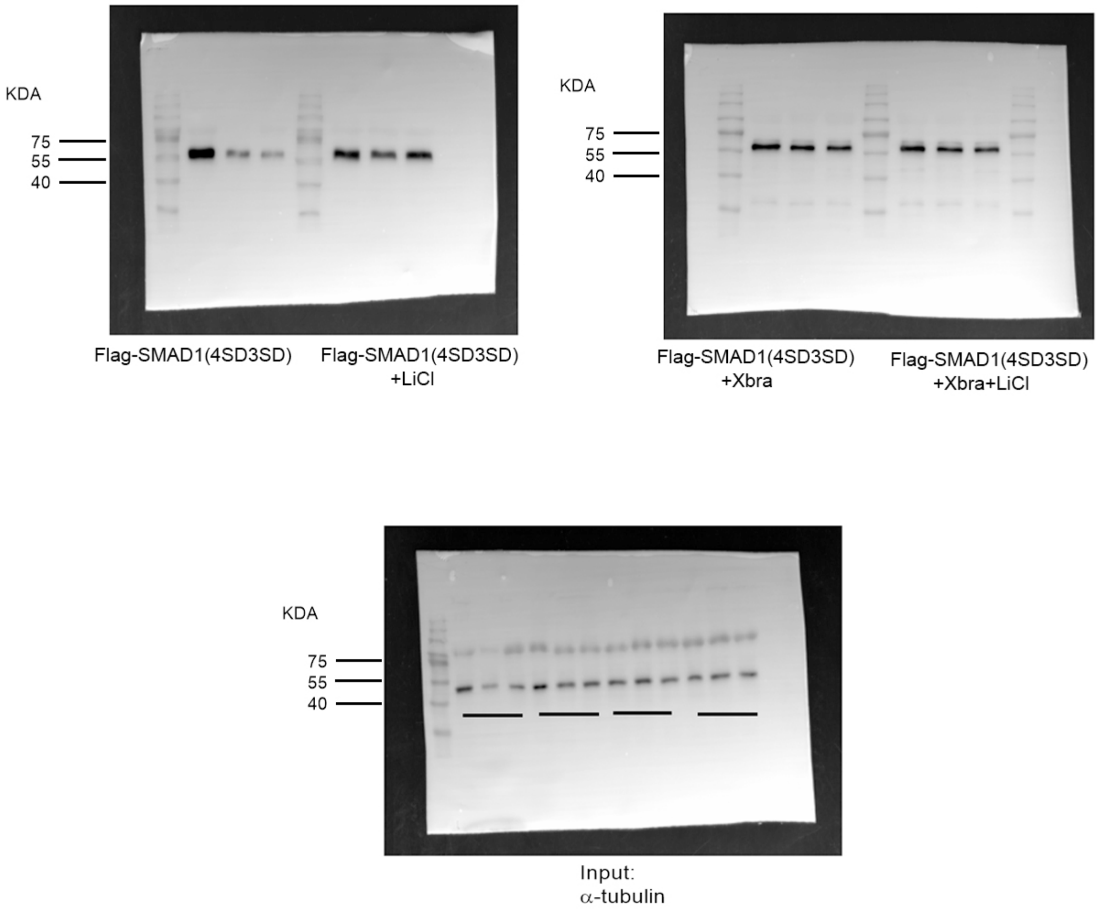
KDA
75
55
40
Flag-SMAD1(4SD3SD)
Flag-SMAD1(4SD3SD) +LiCl
KDA
75
55
40
Flag-SMAD1(4SD3SD) +Xbra
Flag-SMAD1(4SD3SD) +Xbra+LiCl
KDA
75
55
40
Input:
α-tubulin

Figure: 5

B

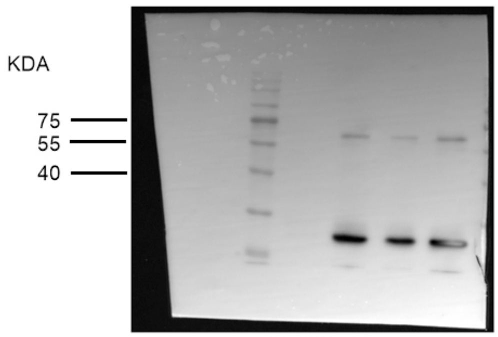

IP:SMAD1
WB:Myc

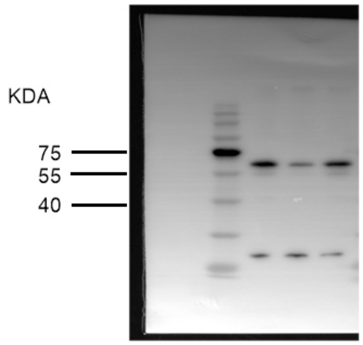

IP:SMAD1
WB:MYC (Replicate)

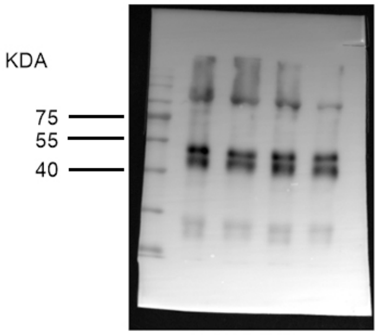

IP:SMAD1
WB:SMAD1

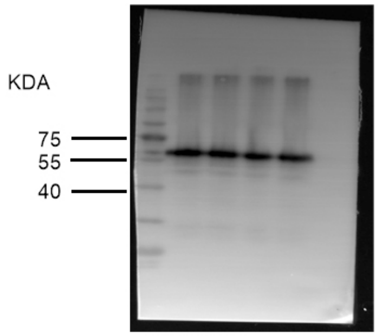

IP:SMAD1
WB:SMAD1 (Replicate)

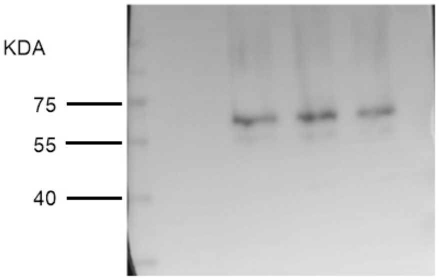

Input:Myc

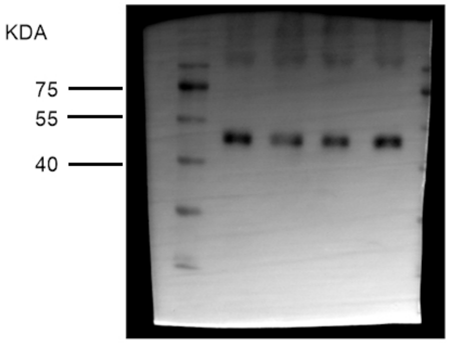

Input:
α-tubulin

C

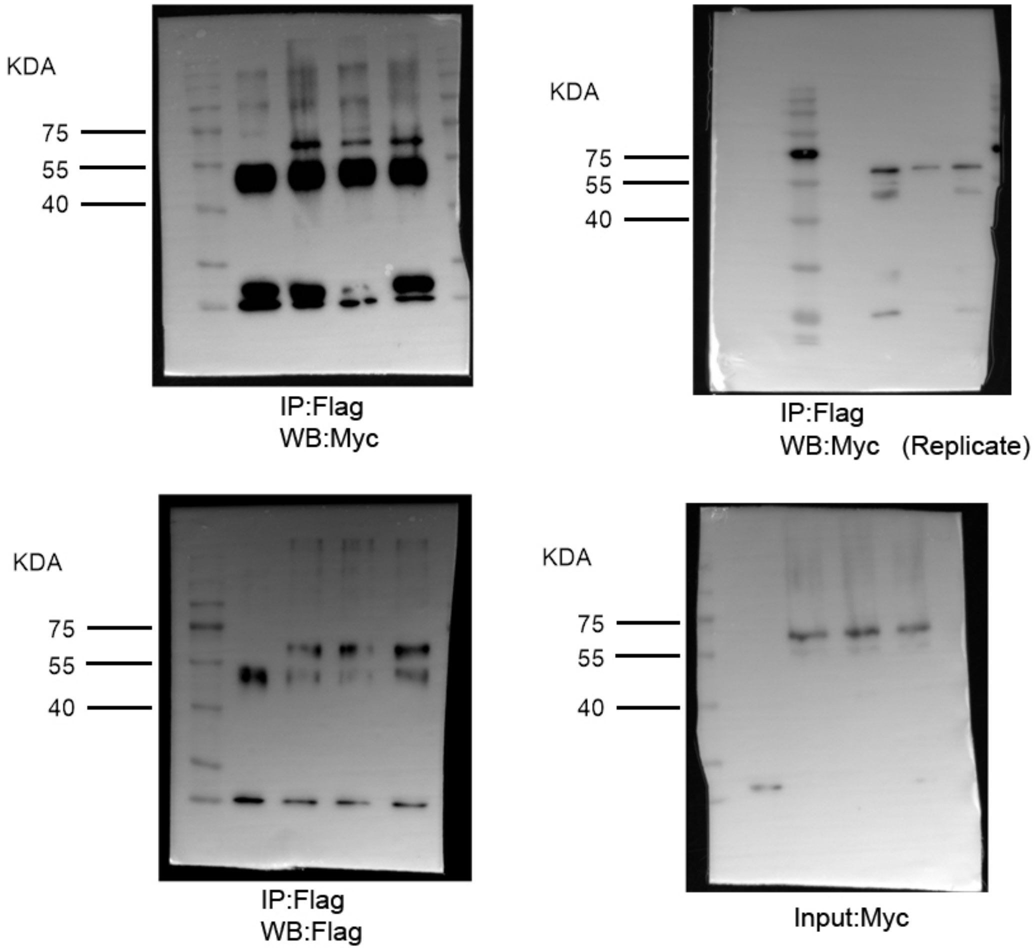
KDA
75
55
40
IP:Flag
WB:Myc
KDA
75
55
40
IP:Flag
WB:Myc (Replicate)
KDA
75
55
40
IP:Flag
WB:Flag
KDA
75
55
40
Input:Myc
